# First person – Koray Ütkür

**DOI:** 10.1242/dmm.050485

**Published:** 2023-09-22

## Abstract

First Person is a series of interviews with the first authors of a selection of papers published in Disease Models & Mechanisms, helping researchers promote themselves alongside their papers. Koray Ütkür is first author on ‘ DPH1 and DPH2 variants that confer susceptibility to diphthamide deficiency syndrome in human cells and yeast models’, published in DMM. Koray is a PhD student in the lab of Prof. Dr Raffael Schaffrath at Universität Kassel, Kassel, Germany, investigating diphthamide deficiency syndrome in yeast cells as a human disease model.


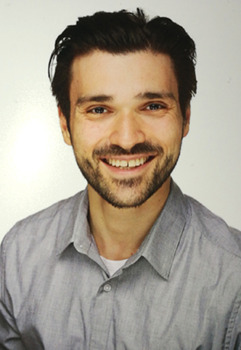


**Koray Ütkür**

**How would you explain the main findings of your paper to non-scientific family and friends?**

There are hereditary diseases that affect life from an early age and come with physical and intellectual impairments. These neurodevelopmental disorders include the very recently discovered diphthamide deficiency syndrome (DDS), the centre of our study. DDS is extremely rare; therefore, very few cases are known to date. The syndrome occurs when both copies of specific genes (*DPH1* and *DPH2*) are not functioning as well as they should due to mutations. The number of uncharacterized *DPH1* and *DPH2* variants, however, is very high and this is where our study comes into place: we have studied a set of potentially harmful variants in human cells and yeast as a model for human disease. Our investigated *DPH1* and *DPH2* variants naturally occur in populations, yet their harmfulness is masked by another intact gene copy (autosomal recessive). The majority of the variants that seemed interesting to us turned out to be prone to triggering DDS. This kind of work is important to help paediatricians and geneticists diagnose patients with DDS.

**What are the potential implications of these results for your field of research?**

Our results expand the number of missense variants within *DPH1* and *DPH2* likely to cause DDS. Our findings are targeted to help DDS diagnosis by increasing the number of variants to be proven either pathogenic or benign. Given that our set of investigated variants is limited, it is likely that more human *DPH1* and *DPH2* variants are causative of DDS. It is also possible that compromises in genes other than *DPH1* and *DPH2* may act in a similar manner, as recently proven for *DPH5*-related diphthamide deficiency.

**What are the main advantages and drawbacks of the experimental system you have used as it relates to the disease you are investigating?**

The cooperativity of both human and yeast systems in providing robust results is our greatest advantage. Our experimental system is based on cell lines of two organisms: human breast cancer cells (MCF7), which are physiologically relevant in mimicking consequences of human diseases, and yeast. Yet, our human assay system includes gene overexpression. Yeast cells, although evolutionarily distant, share gene conservation with human *DPH1* and *DPH2.* Simultaneously, yeast enables implementation of missense mutations on a chromosomal level with minimal genetic/physiological disturbances. I imagine a system without drawbacks to be based on human cells in which one could implement missense mutations on a chromosomal level, ideally including diploid genotypes.

[…] functional outcomes of enzyme variants in human cells and yeast are congruent on a high level even though the organisms are evolutionarily very distant.

**Figure:**
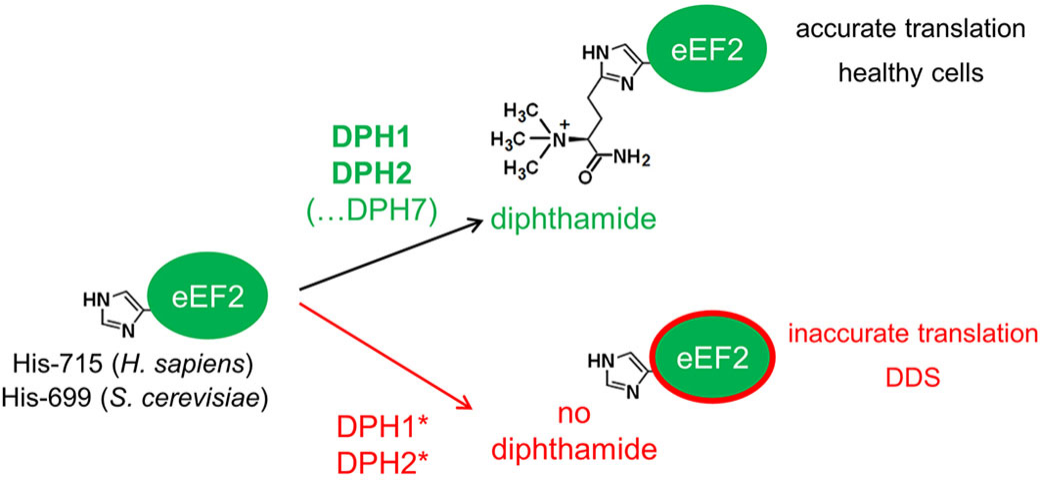
The molecular cause of diphthamide deficiency syndrome (DDS) in human cells and yeast.

**What has surprised you the most while conducting your research?**

For one, I am amazed that functional outcomes of enzyme variants in human cells and yeast are congruent on a high level even though the organisms are evolutionarily very distant. I am additionally excited that we may have obtained further insights into the activation of DPH1 by DPH3. The specifics of our ‘DPH1 stepping stones’ hypothesis are described in our Discussion.

**What do you think is the most significant challenge impacting your research at this time and how will this be addressed over the next 10 years?**

Right now, it is important to facilitate DDS diagnosis and raise awareness for DDS in the first place, given its rarity. The ultimate challenge in DDS-related research is to find and develop a treatment/cure for this hereditary syndrome.

**What changes do you think could improve the professional lives of scientists?**

I have experienced the last few years in research to be very rewarding, yet research positions to be quite limited. On that note, I would wish for increased availability of research grants/stability for scientists. This would attract more scientists to stay in research and accelerate scientific progress.

**What's next for you?**

The field of diphthamide (and its disease relevance) offers a plethora of research questions to be explored. Personally, I am currently in an advanced stage of writing my thesis and I am looking forward to future career chances.
